# Retrotransposon Hypomethylation in Melanoma and Expression of a Placenta-Specific Gene

**DOI:** 10.1371/journal.pone.0095840

**Published:** 2014-04-23

**Authors:** Erin C. Macaulay, Hester E. Roberts, Xi Cheng, Aaron R. Jeffs, Bruce C. Baguley, Ian M. Morison

**Affiliations:** 1 Department of Pathology, Dunedin School of Medicine, University of Otago, Dunedin, New Zealand; 2 Gravida: National Centre for Growth and Development, Auckland, New Zealand; 3 Auckland Cancer Society Research Centre, University of Auckland, Auckland, New Zealand; CEA - Institut de Genomique, France

## Abstract

In the human placenta, DNA hypomethylation permits the expression of retrotransposon-derived genes that are normally silenced by methylation in somatic tissues. We previously identified hypomethylation of a retrotransposon-derived transcript of the voltage-gated potassium channel gene *KCNH5* that is expressed only in human placenta. However, an RNA sequence from this placental-specific transcript has been reported in melanoma. This study examined the promoter methylation and expression of the retrotransposon-derived *KCNH5* transcript in 25 melanoma cell lines to determine whether the acquisition of ‘placental’ epigenetic marks is a feature of melanoma. Methylation and gene expression analysis revealed hypomethylation of this retrotransposon in melanoma cell lines, particularly in those samples that express the placental *KCNH5* transcript. Therefore we propose that hypomethylation of the placental-specific *KCNH5* promoter is frequently associated with *KCNH5* expression in melanoma cells. Our findings show that melanoma can develop hypomethylation of a retrotransposon-derived gene; a characteristic notably shared with the normal placenta.

## Introduction

The human placenta, a globally hypomethylated tissue, is becoming increasingly known for harbouring unmethylated repetitive sequences [1]. The epigenetic activation of repeat sequences in the placenta is demonstrated by the finding that many repetitive elements that are normally silenced by methylation in somatic tissues are transcribed in the placenta [1]–[5]. Of the repeat elements, the *Alu* elements, long interspersed nuclear elements (LINEs), and satellite regions display a reduced level of methylation in the placenta compared to the majority of somatic tissues [6]–[8]. The unique placental expression of these normally silenced repeat elements suggests a functional role for these sequences in the placenta, a role exemplified by the retrovirus-derived gene syncytin (*ERVWE1*), which is essential for trophoblast differentiation during placental development [9], [10].

The loss of DNA methylation at certain repetitive sequences has been shown to be a hallmark in some cancers [11], [12]. Hypomethylation of repetitive elements has been associated with genome instability, a trait that is commonly associated with cancer [12]–[14]. The parallel hypomethylation of repetitive elements in the placenta and in cancer is just one of the many well-documented similarities between placental and cancer cells. These two cell types also share common molecular mechanisms that are thought to regulate their similar proliferative, migratory and invasive phenotypes [15]. The invasion of the trophoblast during placental development is noted to be similar to tumour invasion in cancer [16], [17]. In addition, the co-existence of genetically dissimilar fetal and maternal cells in the placenta echoes the interface of genetically normal and genetically abnormal cancer cells that occurs in neoplasms.

We previously identified a novel retrotransposon-derived transcript of the *KCNH5* gene that is hypomethylated and expressed specifically in the human placenta [18]. The expression of this placental transcript (referred to in this study as p*KCNH5*; previously called transcript 1a) is regulated by a hypomethylated promoter and first exon region that recently arose from a SINE (*AluY*) retrotransposition event. This region is silenced and methylated in all studied non-placental tissues. Data from the Roadmap Epignomics Project (http://www.roadmapepigenomics.org/) show that placenta is the only normal tissue with DNAseI hypersensitivity at the p*KCNH5* promoter, supporting its unique hypomethylated status [19]. From this same source, MeDIP data confirms methylation of the p*KCNH5* promoter in somatic tissues.

Of the six reported human mRNA sequences for *KCNH5* in GenBank (http://www.ncbi.nlm.nih.gov/genbank/), two sequences begin translation using this upstream SINE-derived placental promoter, giving rise to two p*KCNH5* transcripts that differ in their 3′ ends [20]. The remaining four mRNA sequences in GenBank are translated using a downstream promoter, which is unmethylated and used for expression in a wide range of somatic tissues [18]. This downstream promoter gives rise to somatic *KCNH5* transcripts that also differ in their 3′ ends (collectively referred to in this study as s*KCNH5*; previously called transcript 1b). Although “p*KCNH5*” and “s*KCNH5*” are not the current nomenclature for the reported *KCNH5* transcripts, these terms are being used in this study to highlight whether the placental (p) or somatic (s) promoter is being used for *KCNH5* transcription. Based on the reported transcripts, each promoter of *KCNH5* is predicted to give rise to two protein isoforms, resulting in four predicted isoforms for KCNH5. Unexpectedly, one p*KCNH5* mRNA and two EST sequences in Genbank were derived from melanotic melanoma (BC043409, BQ439615 and BX281706).

The *KCNH5* gene (also known as *EAG2*), encodes an *EAG* voltage-gated potassium channel that is involved in the regulation of cell cycle and proliferation [21]. Previous work investigating the oncogenic potential of a closely related *EAG* potassium channel (*EAG1*/*KCNH1*; ∼70% homologous to *KCNH5* [22], [23]) detected expression in several somatic cancer cell lines (including melanoma), normal adult brain and placenta, but not in other normal somatic cells [21]. These expression patterns prompted investigation of *KCNH1* as a potential cancer biomarker [24]. Although the function of *KCNH1* is better understood than that of *KCNH5*, both of these genes are thought to be involved in cell cycle regulation and tumour progression in cancer [21], [25].

Given that the placental-specific transcript of *KCNH5* has been detected in melanoma [26], [27], we aimed to investigate whether its retrotransposon-derived promoter, which is hypermethylated in those studied normal tissues from the body [18], becomes hypomethylated in melanoma. We hypothesised that genes that are hypomethylated and active in the placenta may become inappropriately activated in cancer tissues by similar epigenetic mechanisms.

## Materials and Methods

### Ethics Statement

The samples of human placenta were obtained with written consent under the approval of the Lower South Regional Ethics Committee (New Zealand). The human fetal tissues (brain, liver, heart, stomach, adrenal) were obtained with written consent under the approval of the Otago Ethics Committee (New Zealand). The human adult somatic tissues (brain, kidney, heart, liver, spleen, pancreas, lung and colon) were obtained at autopsy, following informed written consent, under the provisions of Section 3 of the Human Tissue Act 1964 [28]. The New Zealand melanoma (NZM) cell lines used in this study were generated from surgical samples of metastatic melanoma, obtained with written consent from all patients under the guidelines and specific approval of the Auckland Area Health Board Ethics Committee (New Zealand).

### Sample Collection

The 25 human melanoma (NZM) cell lines used in this study were generated from American Joint Committee on Cancer (AJCC) Stage III or IV tumours of consenting patients as described previously [29]–[31]. Phenotype data was available for ten of the melanoma cell lines; categorised as either “more invasive” or “less invasive” (previously referred to as Motif 1 and Motif 2 [32]). The more invasive cells (n = 5) are NZM09, NZM11, NZM22, NZM40, NZM52 and the less invasive cells (n = 5) are cell lines NZM06, NZM12, NZM15, NZM42, NZM45. In addition to the 25 melanoma cell lines, RNA and DNA from three patients' metastatic tumours were included in this study (samples NZM53T, NZM58T, and NZM62T). Two of the metastatic tumour samples (NZM53T and NZM58T) also had derivative cell lines in this study (NZM53 and NZM58).

The samples of human placenta consisted of 31 first-trimester placentas and one term placenta. The 31 first-trimester human placenta samples were collected and processed as described previously [18]. Six first-trimester placenta samples were of 35–41 days gestation, 12 samples were of 42–48 days gestation, and 13 samples were of 49–55 days gestation. The three pooled sections from one human term placenta (maternal facing, middle and fetal facing) were processed as described previously [18]. The human fetal tissues (brain, liver, heart, stomach, adrenal) were previously collected from medically terminated pregnancy tissues. The adult somatic tissues (brain, kidney, heart, liver, spleen, pancreas, lung and colon) were collected at autopsy from consenting patients on the basis that the tissues were non-diseased (as per medical records). Peripheral blood was collected from healthy donors.

### Gene Expression Analysis

RNA was extracted from melanoma cell lines and tumour tissue by using a combined Trizol/column clean-up protocol as described in [24]. For non-melanoma samples, RNA was extracted from 50 mg of frozen tissue (placenta, fetal or adult tissues) using the PureLink Micro-to-Midi Total RNA Purification System (Invitrogen, Cat #12183-018). Complementary DNA was made from 300 ng RNA by using the Applied Biosystems High Capacity RNA-to-cDNA Kit (Applied Biosystems, Cat #4387406) following the manufacturer's protocol. To test cDNA quality, a Reverse-Transcriptase-PCR (RT-PCR) was performed on all samples for the beta-2-microglobulin (*B2M*) housekeeping gene. Melanoma samples were then screened for *KCNH5* expression using the RT-PCR protocol described previously [18]. Primer sequences for RT-PCRs are listed in Table S1. The presence of a 423 bp product confirmed expression using the p*KCNH5* promoter and the presence of a 369 bp amplicon confirmed expression using the s*KCNH5* promoter. RT-negative and no-template (water) controls were used in PCRs to exclude non-specific amplification and genomic contamination. PCR product was then purified from all samples in both p*KCNH5* and p*KCNH5* RT-PCRs using QIAquick PCR purification kit (Qiagen, Cat #28106) following the manufacturer's protocol. The purified PCR product was sequenced using the forward (transcript-specific) primers by the Genetic Analysis Services (Department of Anatomy, University of Otago) to determine which *KCNH5* promoter was used for transcription in each melanoma sample. Sequences were aligned to the reported p*KCNH5* and s*KCNH5* sequences [33] and alignment within the first 114 bp of p*KCNH5* and 61 bp of s*KCNH5* confirmed the specific gene product. Beyond this point the sequence of the two transcripts is the same (and continues until exon 7).

### Quantitative Gene Expression Analysis

Primers for RT-qPCR were designed to the promoter and unique exons 1 and 2 of p*KCNH5* using the IDT RealTime PCR Custom Assay Design online tool (http://eu.idtdna.com/Scitools/Applications/RealTimePCR). Primer sequences are listed in Table S2. Transcript abundance was measured using Power SYBR® Green PCR Master Mix (Applied Biosystems, Cat #4367659) with ROX reference dye on an ABI 7300 Real-Time PCR System. Standard curves for p*KCNH5* and two reference genes, *GNB2L1* and *RPL13A* were generated to assess primer efficiency. Relative transcript abundance was calculated by using qBase+ software [34] with target-specific amplification efficiency correction and normalisation to the *GNB2L1* and *RPL13A* reference genes. For each assay, reactions were performed in triplicate and Ct values were excluded if they differed by more than 0.55 Ct. Relative transcript abundance of p*KCNH5* was scaled to the sample with the lowest transcript abundance (NZM61).

### Sequenom MassARRAY EpiTYPER Analysis

DNA was extracted from melanoma cell lines and tumour tissue by using a Genomic DNA Isolation Kit (Norgen Biotek Cat. #24700) according to the manufacturer's instructions. Matched DNA samples were available for all but three of the melanoma RNA samples that were analysed for p*KCNH5* expression (NZM07, NZM7.4 and NZM57). DNA was extracted from placenta, fetal and somatic tissues using the QIAamp DNA Mini Kit (Qiagen, Cat. #51306) according to the manufacturer's protocol. A water sample was included as a negative control. Samples of melanoma DNA, pooled placenta, fetal and somatic tissues were bisulfite converted using Zymo Research EZ DNA Methylation Kit (Zymo Research, Cat. #D5002) following an alternative cycling protocol: 95°C for 30 seconds, 50°C for 15 minutes for 20 cycles. PCR was performed directly on bisulphite converted DNA according to the manufacturer's protocol (Sequenom – San Diego, CA). Primers for p*KCNH5* and s*KCNH5* were designed using the EpiTyper software (Sequenom) and are listed in Table S3. Sequenom data was analysed using the EpiTYPER software (Sequenom). Mean methylation was calculated for the whole amplicon. Technical replicates were performed on three samples: pooled first trimester placenta, pooled somatic tissues and pooled fetal tissues.

## Results

### Gene Expression Analysis of p*KCNH5* in Melanoma

**Figure 1:**
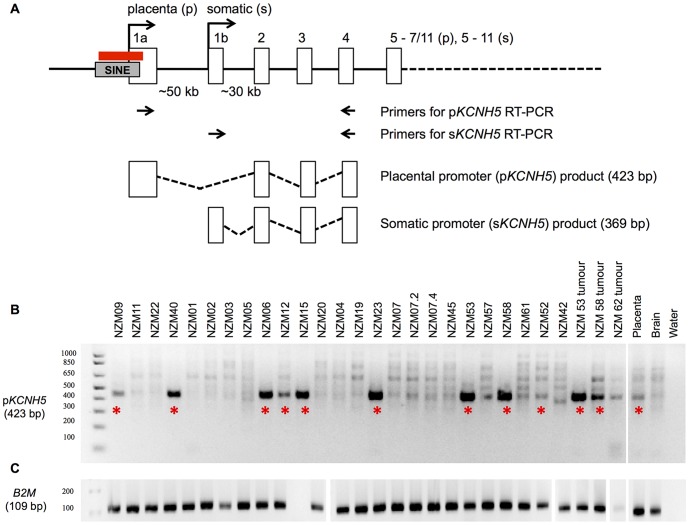
End-point RT-PCR screening for p*KCNH5* in melanoma. **A**. Genomic map of *KCNH5* showing transcripts that use the placental promoter (p*KCNH5*) and transcripts that use the somatic promoter (s*KCNH5*). Transcripts are different in their first exons and alignment starts at exon two (and continues until exon 7). The grey box shows the SINE retrotransposon and the red line denotes the region of analysed methylation. **B**. Detection of p*KCNH5* RT-PCR product (423 bp) by agarose gel electrophoresis. The positive (placenta) and negative (brain) controls confirmed specific presence and absence of the p*KCNH5* product, respectively. All samples were sequenced and red asterisks indicate the 11 melanoma samples that were confirmed to use the placental promoter and express p*KCNH5*, as well as the positive control sample of placenta. **C**. Screening cDNA for the internal control *B2M* gene (109 bp product). The varying product intensities are likely to result from differences in *B2M* mRNA template produced by each melanoma cell line. Sample NZM15 consistently displayed negligable *B2M* expression yet was positive for p*KCNH5* expression.

*KCNH5* expression was examined by RT-PCR to determine whether melanoma samples used the placental promoter for *KCNH5* transcription. In the p*KCNH5* RT-PCR, the presence of a 423 bp PCR product was detected in 11 of the 25 melanoma samples (Figure 1B). Sequencing all samples from this PCR confirmed the use of the placental *KCNH5* promoter in the 11 melanoma samples. The quality of cDNA was confirmed by RT-PCR of the *B2M* gene (Figure 1C).

### Methylation Analysis of p*KCNH5* in Melanoma

**Figure 2:**
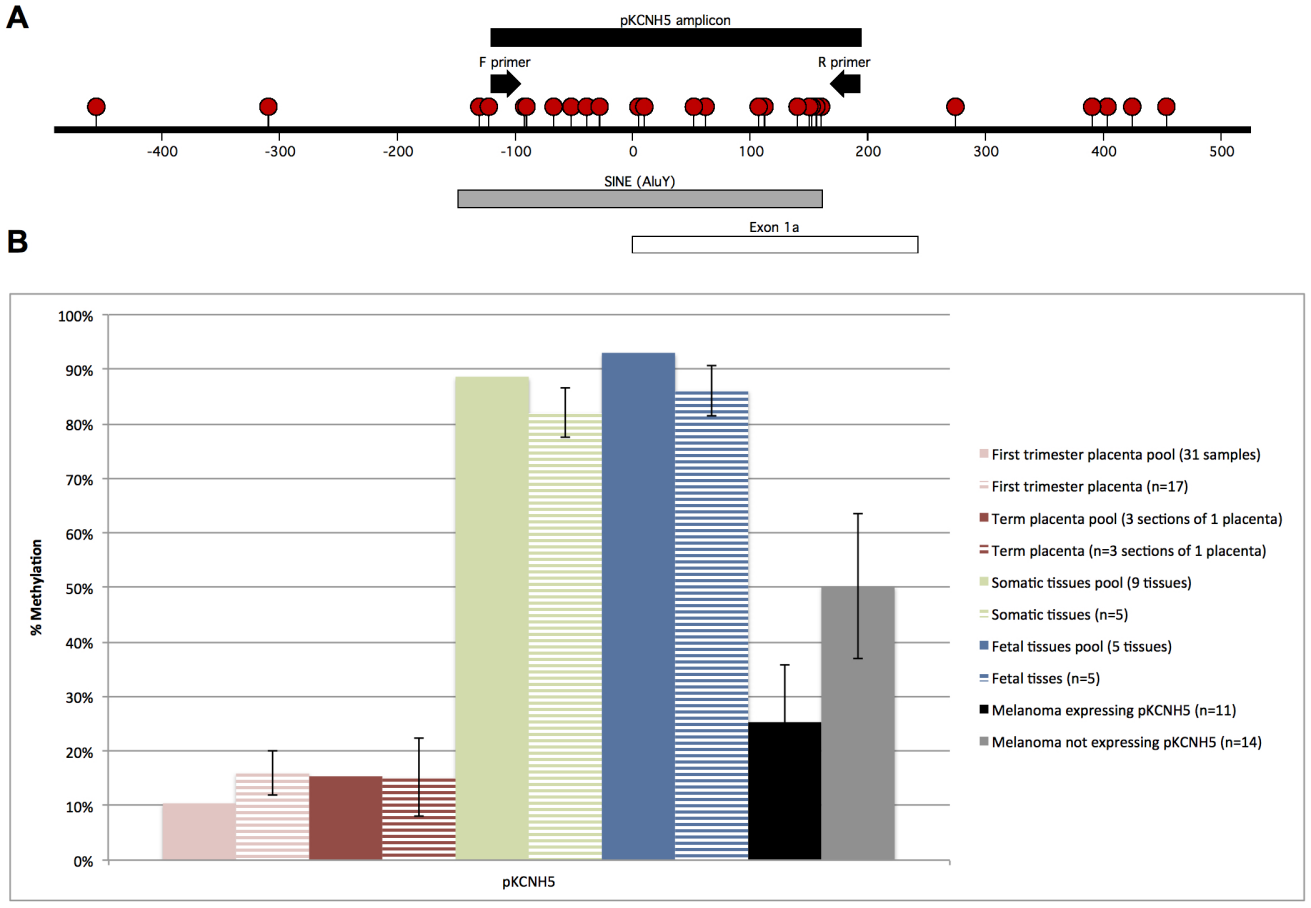
Mean promoter methylation for p*KCNH5*. **A**. Genomic map of the p*KCNH5* amplicon that was examined by Sequenom. Coordinates refer to the genomic location with respect to the p*KCNH5* transcription start site. Red circles represent individual CpG sites. Black arrows represent primers used to amplify the 313 bp product, which contained 17 CpG sites that were analysed for methylation (all of which are located within the SINE (*AluY*) element). **B**. Columns represent mean CpG methylation for the amplicon. Solid bars represent Sequenom data from the present study and lined bars represent previously published Sequenom data [18]. In the present study, somatic and fetal tissues were quantified as pools of DNA (somatic pool: adult brain, kidney, heart, liver, spleen, pancreas, lung, colon and peripheral blood; fetal pool: fetal brain, liver, heart, stomach, and adrenal). In the previous study, somatic tissues (brain, kidney, heart, liver, spleen) and fetal tissues (brain, liver, heart, kidney and adrenal) were quantified as individual tissues; the lined bars represent mean methylation of these samples. Based on end-point p*KCNH5* RT-PCR data (confirmed by sequencing), 11 melanoma samples express p*KCNH5* (NZM09, NZM40, NZM06, NZM12, NZM15, NZM23, NZM53, NZM58, NZM52, NZM53T and NZM58T) and 17 samples did not express p*KCNH5*. Error bars represent the 95% confidence interval of the mean.

Sequenom analysis was used to quantify the level of retrotransposon methylation within the promoter region of p*KCNH5* (Figure 2A). Mean p*KCNH5* methylation was lower in melanoma samples that expressed the placental transcript (25%) and higher in melanoma samples that did not express the transcript (50%) (Figure 2B). The low levels of methylation in first trimester placenta (11%) and term placenta (15%) and high levels of methylation in pooled somatic tissues (89%) and pooled fetal tissues (93%) are consistent with our previously published results [18] (which are included in Figure 2).

### Quantitative Expression Analysis of p*KCNH5* in Melanoma

**Figure 3:**
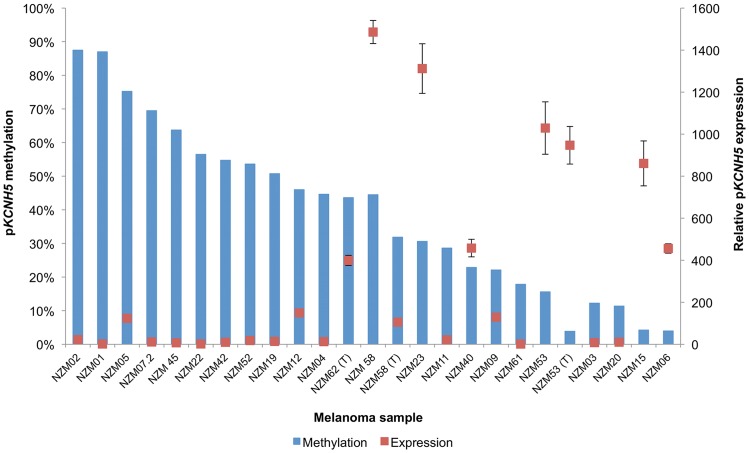
Mean promoter methylation and relative expression of p*KCNH5* in melanoma cell lines. Average promoter methylation (%) is depicted on the left-hand axis and displayed as blue columns. p*KCNH5* expression (relative to the lowest expressing cell line; NZM61) is depicted on the right-hand axis and shown as red circles. Error bars represent the standard error of the mean. Samples labeled “(T)” refer to samples from metastatic tumour RNA/DNA (NZM53T, NZM58T and NZM62T).

**Figure 4:**
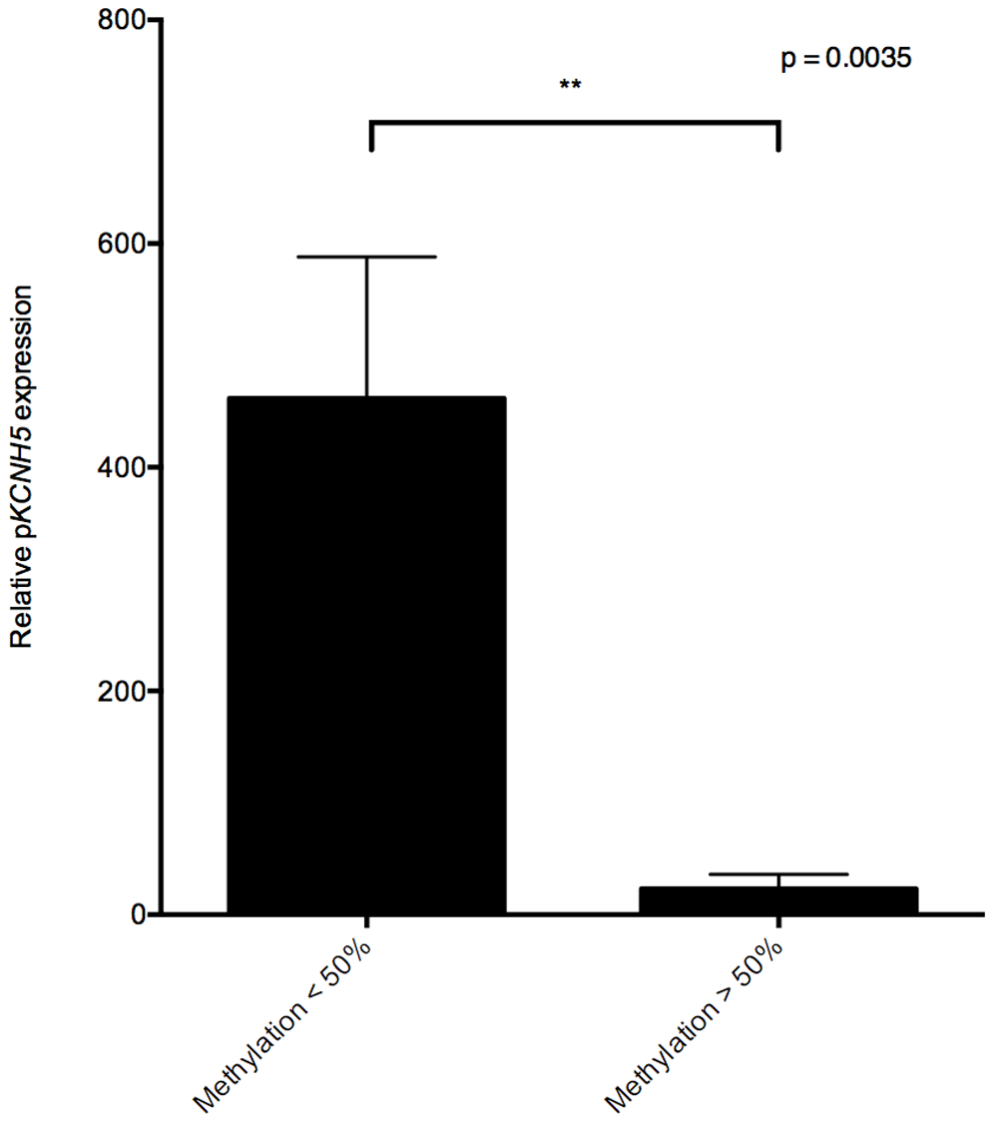
Relative p*KCNH5* expression in melanoma cells based on degree of promoter methylation. Columns represent the mean relative p*KCNH5* expression value for the 25 melanoma cell lines (grouped by promoter methylation status of <50% or >50%). Expression data for 16 cell lines is displayed in the <50% methylation column, and expression data for nine cell lines is displayed in the >50% methylation column. An unpaired T-test with Welch's correction was performed, yielding a p-value of 0.0035. Error bars represent the standard error of the mean.

Quantitative expression analysis of p*KCNH5* in melanoma showed an inverse relationship between p*KCNH5* expression and promoter methylation (Figure 3). Not all hypomethylated tumour samples showed expression of p*KCNH5*, but there appears to be a threshold at which p*KCNH5* hypomethylation may become permissive of expression, i.e., less than approximately 50% methylation. Indeed, p*KCNH5* transcript expression in cell lines with above 50% promoter methylation was significantly lower than those with below 50% (p = 0.0017; Figure 4). Additional analysis of individual CpG sites in the p*KCNH5* promoter did not reveal a specific CpG site whose methylation was more predictive of expression than that of the promoter as a whole.

### Gene Expression and Methylation Analysis of s*KCNH5* in Melanoma

Our previous comparison of placental and somatic tissues suggested that transcription of p*KCNH5* is independent of that from the s*KCNH5* promoter. For s*KCNH5*, RT-PCR followed by sequencing revealed that the somatic promoter was expressed in five of the 25 melanoma cell lines (NZM01, NZM06, NZM40, NZM52, NZM53) (Figure S1); four of which also used the placental promoter (NZM06, NZM40, NZM52, NZM53). The negative (placenta) and positive (brain) controls showed presence and absence of the s*KCNH5* product, respectively. The association between p*KCNH5* and s*KCNH5* expression is shown in Figure S3A.

Mean methylation of the s*KCNH5* promoter was found to be low in nearly all examined tissues including melanoma (Figure S2). There was no correlation between the methylation of p*KCNH5* and s*KCNH5* promoters in melanoma samples (Figure S3B). The association between s*KCNH5* methylation and s*KCNH5* expression in melanoma is shown in Figure S3C.

## Discussion

The *EAG* class of ion channels is overexpressed in many cancers [35], [36]. It is suggested that they regulate cell proliferation through control of the entry of cells into the G1 phase of the cell cycle [37], [38]. Therefore, molecular changes to *EAG* channels may result in dysregulation of cellular proliferation, one of the hallmarks of carcinogenesis. In this study, the expression of p*KCNH5* in melanoma cells correlates with hypomethylation of the promoter region, suggesting that this epigenetic change may accompany the expression of p*KCNH5* which contributes to the oncogenic change in melanocytes [39], [40].

DNA hypomethylation in melanoma has been associated with aberrant gene expression and the unsilencing of normally methylated repeat regions. Studies have shown that hypomethylation in melanoma correlates with activation and expression of some of the cancer testis (CT) genes [41], [42]. CT genes encode protein antigens that are normally expressed in adult testicular germ cells (and not in somatic tissues) [43], [44]. Given the number of hypomethylated (and aberrantly activated) CT genes in melanoma, they are now being studied as potential targets for slowing melanoma progression [42], [44]. In addition, hypomethylation of the non-coding retrotransposon sequence, long interspersed nuclear element-1 (LINE1), was identified as a feature of melanoma, with the degree of LINE1 hypomethylation positively correlated with melanoma progression [45].

In this study, the expression of p*KCNH5* is significantly higher in melanoma cell lines that have lower levels of promoter methylation. Hypomethylation alone does not guarantee increased gene expression, but can act permissively to allow gene expression. There were no differences in promoter methylation or p*KCNH5* expression between the “more invasive” and “less invasive” melanoma cell lines (data not shown). This suggests that p*KCNH5* is not involved in invasion, which was not surprising given that *KCNH5* is thought to be a regulator of cell proliferation instead of invasion [37], [38]. Although the use of cultured melanoma cell lines presents a risk for culture-induced epigenetic changes, the small differences in methylation (12% and 13%) between the tumour samples (NZM53T and NZM58T) and their derivative cell lines (NZM53 and NZM58) suggests that the cell lines did not acquire significant epigenetic aberrations in culture.

Similar to our previous findings [18], methylation of the somatic *KCNH5* promoter (s*KCNH5*) was low in all of the examined tissues. Expression of s*KCNH5*, the promoter of which is not derived from a retrotransposon, is not associated with p*KCNH5* expression in melanoma samples (Figure S3A). The methylation of s*KCNH5* is also not related to p*KCNH5* methylation in melanoma (Figure S3B). Importantly, we and others have found that primary adult melanocytes do not express either transcript of *KCNH5* (Supplementary Figure 4 in [18] and data from the UCSC browser [26]). According to data from the Roadmap Epigenomics Project, melanocytes show no DNase1 sensitivity at the s*KCNH5* promoter, suggesting a condensed chromatin state (not transcriptionally active) at that site [19]. Thus the finding that melanoma samples express the placental transcript of *KCNH5* is notable given that healthy (non-cancerous) melanocytes do not express either transcript of this gene.

We have previously demonstrated that the retrotransposon-derived transcript of *KCNH5* is expressed exclusively in the human placenta. We detected high levels of methylation in its promoter and did not detect expression in any of the somatic tissues (brain, kidney, heart, liver and spleen) [18]. Therefore, the expression of p*KCNH5* and its coordinate hypomethylation in melanoma may reflect a tumour-associated epigenetic change. Hypomethylation and expression of many retrotransposon-derived sequences in the placenta is assumed to contribute to the invasive features of the primate hemochorial placenta [46], [47]. We speculate that hypomethylation in melanoma cells may involve mechanisms similar to those in the placenta and contribute to their malignant phenotype. Analysis of other retrotransposon-derived promoters in melanoma may provide further insight into hypomethylation as an event that is part of the cancer transformation pathway.

## Supporting Information

Figure S1 **End-point RT-PCR screening for s** ***KCNH5*** **in melanoma.** **A**. Detection of s*KCNH5* product by agarose gel electrophoresis. All samples were sequenced and red asterisks indicate the five melanoma samples that were confirmed to use the somatic promoter (NZM01, NZM06, NZM40, NZM52, NZM53) and express s*KCNH5* (369 bp). **B**. RT-negative samples confirm specificity of the s*KCNH5* RT-PCR.
(TIFF)

Figure S2 **Mean promoter methylation for s** ***KCNH5*** **.** **A**. Genomic map of s*KCNH5* amplicon that was examined by Sequenom. Coordinates refer to the genomic location with respect to the s*KCNH5* transcription start site. Red circles represent individual CpG sites. Black arrows represent primers used to amplify the 445 bp product, which contained 38 CpG sites that were analysed for methylation. **B**. Columns represent mean CpG methylation for the amplicon. Solid bars represent Seqeunom data from the present study and lined bars represent previously published Sequenom data [18]. Error bars represent the 95% confidence interval.
(TIFF)

Figure S3 **Relative p** ***KCNH5*** **expression compared to presence or absence of s** ***KCNH5*** **expression in melanoma.** **A**. Expression of p*KCNH5* was not related to expression of s*KCNH5*. Data points represent relative p*KCNH5* expression values (from qRT-PCR) for the 25 melanoma cell lines, grouped by presence or absence of s*KCNH5* expression. An unpaired T-test with Welch's correction was performed, yielding a p-value of 0.384. **B**. Methylation of p*KCNH5* was not related to s*KCNH5*. Data points represent p*KCNH5* and s*KCNH5* methylation values for the 25 melanoma cell lines. A linear regression analysis was performed, yielding a p-value of 0.335. **C**. The relationship between s*KCNH5* expression and methylation. Data points represent s*KCNH5* methylation and expression values, grouped by presence or absence of s*KCNH5* expression (based on sequecning of end-point RT-PCR product). An unpaired T-test with Welch's correction was performed, yielding a borderline-significant p-value of 0.029, which is largely attributable to a single outlying point (a non-expressing sample with 60% methylation).
(TIFF)

Table S1 **Primer sequences for end-point RT-PCR gene expression analysis of p** ***KCNH5*** **and** ***sKCNH5*** **in melanoma cell lines.**
(DOCX)

Table S2 **Primer sequences for quantitative RT-PCR gene expression analysis of p** ***KCNH5*** **in melanoma cell lines.**
(DOCX)

Table S3 **Primer sequences for Sequenom promoter methylation analysis of p** ***KCNH5*** **and s** ***KCNH5*** **in melanoma cell lines.**
(DOCX)
