# Supplementary figures and images for: Retrotransposon Hypomethylation in Melanoma and Expression of a Placenta-Specific Gene
Source: PLoS One. 2014 Apr 23;9(4):e95840. doi: 10.1371/journal.pone.0095840 (PMC3997481; doi:10.1371/journal.pone.0095840)

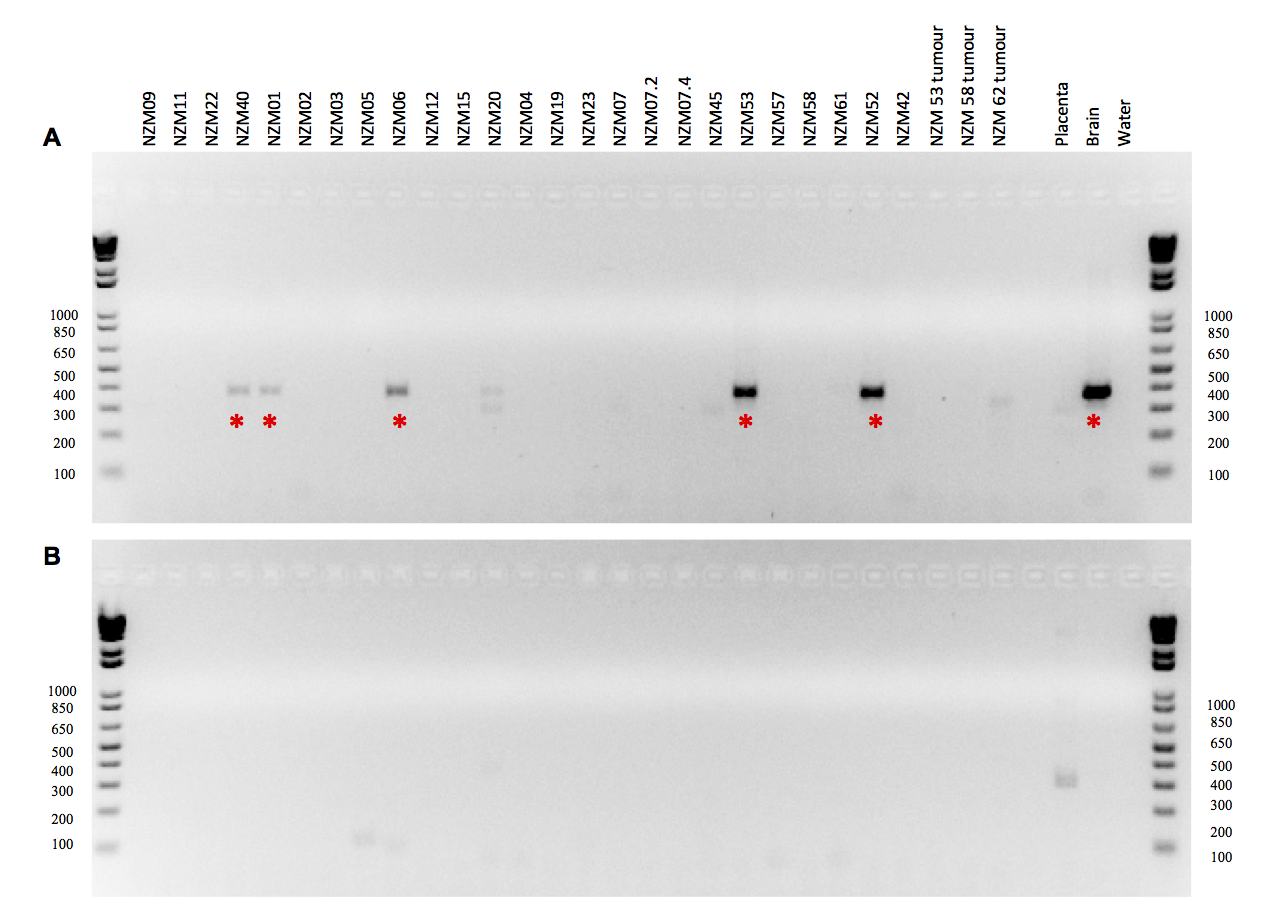

Supplement: Figure S1 — End-point RT-PCR screening for s KCNH5 in melanoma. A. Detection of sKCNH5 product by agarose gel electrophoresis. All samples were sequenced and red asterisks indicate the five melanoma samples that were confirmed to use the somatic promoter (NZM01, NZM06, NZM40, NZM52, NZM53) and express sKCNH5 (369 bp). B. RT-negative samples confirm specificity of the sKCNH5 RT-PCR. (TIFF) [file pone.0095840.s001.tiff]

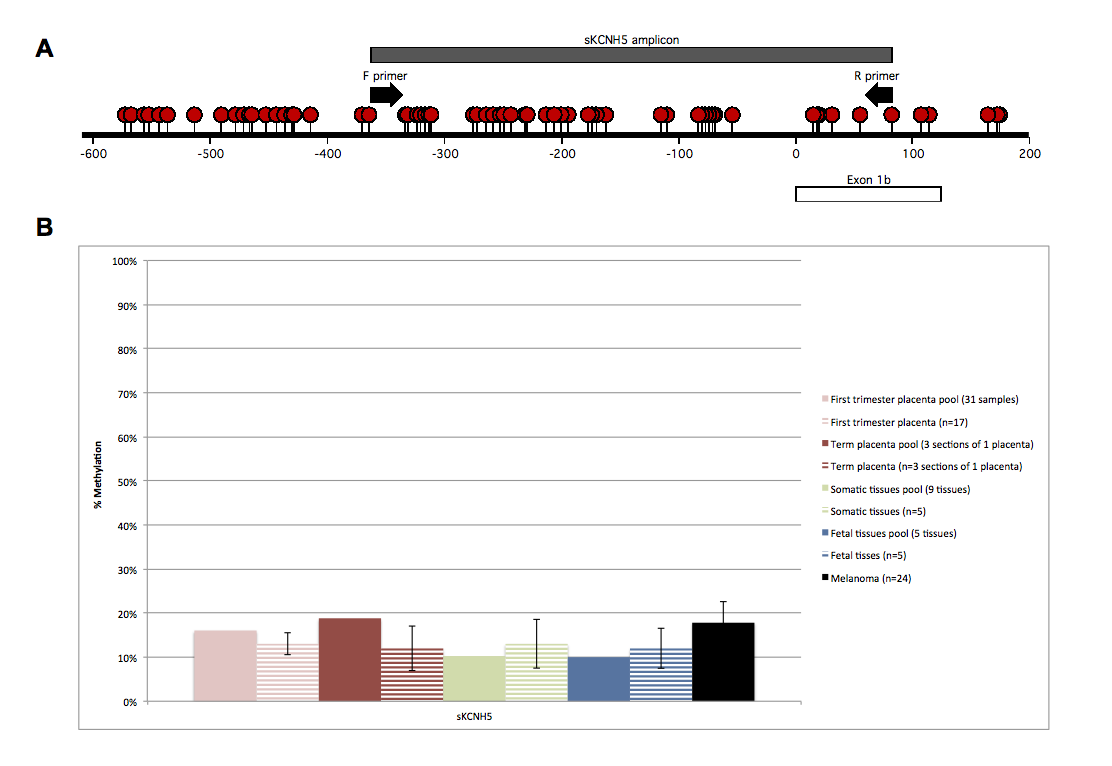

Supplement: Figure S2 — Mean promoter methylation for s KCNH5 . A. Genomic map of sKCNH5 amplicon that was examined by Sequenom. Coordinates refer to the genomic location with respect to the sKCNH5 transcription start site. Red circles represent individual CpG sites. Black arrows represent primers used to amplify the 445 bp product, which contained 38 CpG sites that were analysed for methylation. B. Columns represent mean CpG methylation for the amplicon. Solid bars represent Seqeunom data from the present study and lined bars represent previously published Sequenom data [18]. Error bars represent the 95% confidence interval. (TIFF) [file pone.0095840.s002.tiff]

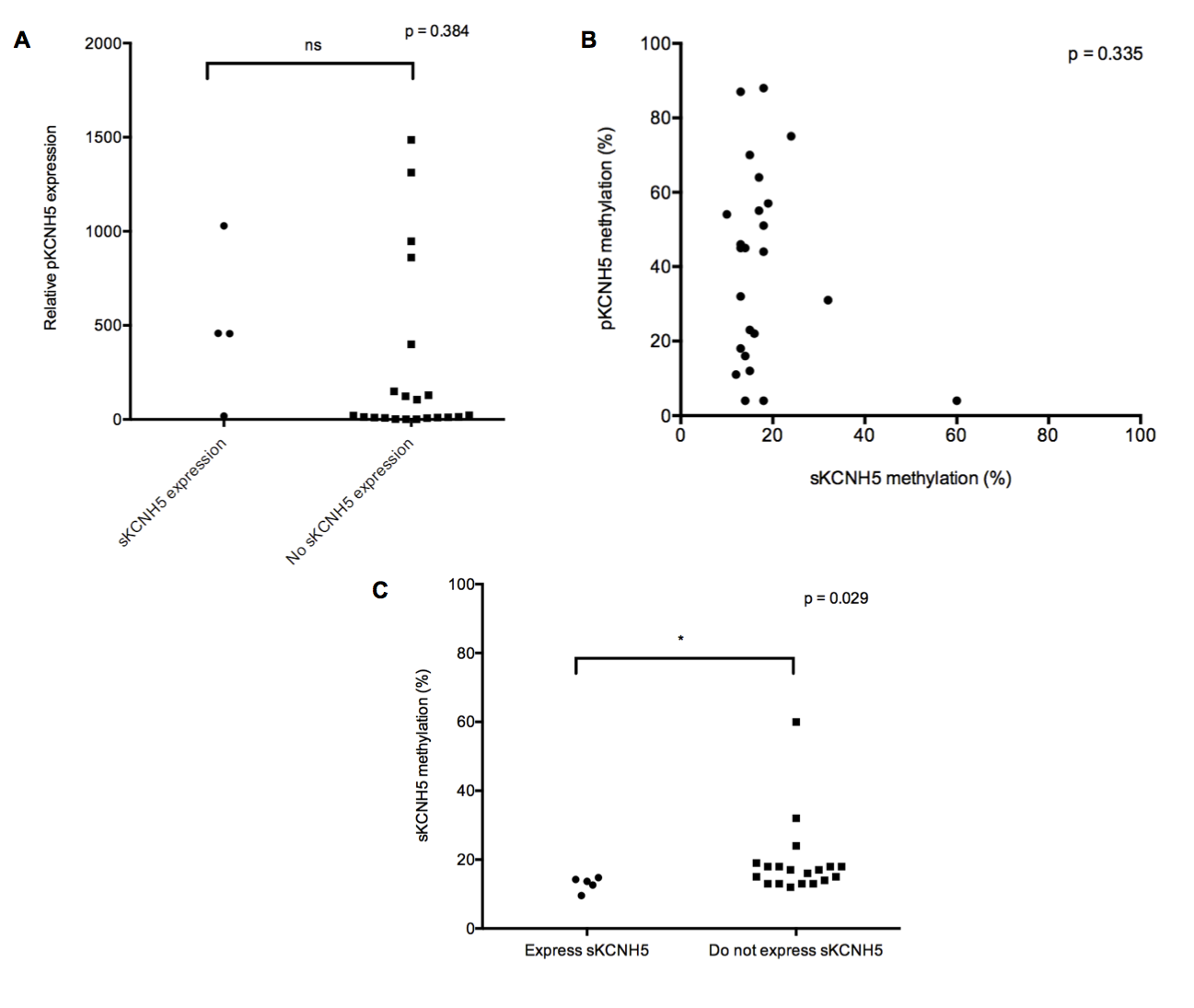

Supplement: Figure S3 — Relative p KCNH5 expression compared to presence or absence of s KCNH5 expression in melanoma. A. Expression of pKCNH5 was not related to expression of sKCNH5. Data points represent relative pKCNH5 expression values (from qRT-PCR) for the 25 melanoma cell lines, grouped by presence or absence of sKCNH5 expression. An unpaired T-test with Welch's correction was performed, yielding a p-value of 0.384. B. Methylation of pKCNH5 was not related to sKCNH5. Data points represent pKCNH5 and sKCNH5 methylation values for the 25 melanoma cell lines. A linear regression analysis was performed, yielding a p-value of 0.335. C. The relationship between sKCNH5 expression and methylation. Data points represent sKCNH5 methylation and expression values, grouped by presence or absence of sKCNH5 expression (based on sequecning of end-point RT-PCR product). An unpaired T-test with Welch's correction was performed, yielding a borderline-significant p-value of 0.029, which is largely attributable to a single outlying point (a non-expressing sample with 60% methylation). (TIFF) [file pone.0095840.s003.tiff]
