# Supplementary material for: Retrotransposon Hypomethylation in Melanoma and Expression of a Placenta-Specific Gene
Source: PLoS One. 2014 Apr 23;9(4):e95840. doi: 10.1371/journal.pone.0095840 (PMC3997481; doi:10.1371/journal.pone.0095840)
Supplement: Table S1 — Primer sequences for end-point RT-PCR gene expression analysis of p KCNH5 and sKCNH5 in melanoma cell lines. (DOCX) [file pone.0095840.s004.docx]

**Table S1.** Primer sequences for end-point RT-PCR gene expression analysis of p*KCNH5* and *sKCNH5* in melanoma cell lines.

| **Gene Target** | **Primer Sequence** | **Amplicon size (bp)** |
| --- | --- | --- |
| **p*KCNH5*** | F- CTGGGATTACAGGCGTGAGC | 423 |
|  | R- AGTACACAGGAACAAGACCAC |  |
| **s*KCNH5*** | F- AAGAGAGGGCTGGTGGCACC | 369 |
|  | R- AGTACACAGGAACAAGACCAC |  |
| ***B2M*** | F- GAGTATGCCTGCCGTGTG | 109 |
|  | R- AATCCAAATGCGGCATCT |  |
