# Supplementary material for: Retrotransposon Hypomethylation in Melanoma and Expression of a Placenta-Specific Gene
Source: PLoS One. 2014 Apr 23;9(4):e95840. doi: 10.1371/journal.pone.0095840 (PMC3997481; doi:10.1371/journal.pone.0095840)
Supplement: Table S2 — Primer sequences for quantitative RT-PCR gene expression analysis of p KCNH5 in melanoma cell lines. (DOCX) [file pone.0095840.s005.docx]

**Table S2.** Primer sequences for quantitative RT-PCR gene expression analysis of p*KCNH5* in melanoma cell lines.

| **Gene Target** | **Primer Sequence** | **Amplicon size (bp)** |
| --- | --- | --- |
| **p*KCNH5*** | F-AACAAAATCCAACACATAAAGATTCAC | 147 |
|  | R-CGTCAGCTCGATGATATCCAG |  |
| ***RPL13A*** | F-CCTGGAGGAGAAGAGGAAAGAGA | 126 |
|  | R-TTGAGGACCTCTGTGTATTTGTCAA |  |
| ***GNB2L1*** | F-CACAACGGGCACCACCAC | 138 |
|  | R-CACACACCCAGGGTATTCCAT |  |
