# Supplementary material for: Retrotransposon Hypomethylation in Melanoma and Expression of a Placenta-Specific Gene
Source: PLoS One. 2014 Apr 23;9(4):e95840. doi: 10.1371/journal.pone.0095840 (PMC3997481; doi:10.1371/journal.pone.0095840)
Supplement: Table S3 — Primer sequences for Sequenom promoter methylation analysis of p KCNH5 and s KCNH5 in melanoma cell lines. (DOCX) [file pone.0095840.s006.docx]

**Table S3.** Primer sequences for Sequenom promoter methylation analysis of p*KCNH5* and s*KCNH5* in melanoma cell lines.

| **Gene Target** | **Primer Sequence** | **Amplicon size (bp)** |
| --- | --- | --- |
| **p*KCNH5*** | F- TTTGTTTTTTAGGTTGGAGTGTAGT | 313 |
|  | R- AATATCTTTCATTTACTCAAAAATT |  |
| **s*KCNH5*** | F- TTTTTTAGTAGGGGGAGAGATGTAGA | 445 |
|  | R- ATACCACCAACCCTCTCTTACC |  |
